# Optimal Feeding Frequency for Captive Hawksbill Sea Turtle (*Eretmochelys imbricata*)

**DOI:** 10.3390/ani11051252

**Published:** 2021-04-26

**Authors:** Suthep Jualaong, Hirun Kanghae, Karun Thongprajukaew, Suktianchai Saekhow, Natthida Amartiratana, Piyanan Sotong

**Affiliations:** 1 Marine and Coastal Resources Research Center, The Eastern Upper Gulf of Thailand, Rayong 21170, Thailand; sutep.emcor@hotmail.com; 2 Phuket Marine Biological Center, Phuket 83000, Thailand; kanghae_h@hotmail.com (H.K.); natthida.a@outlook.com (N.A.); piyanan1995@hotmail.com (P.S.); 3 Division of Health and Applied Sciences, Faculty of Science, Prince of Songkla University, Songkhla 90112, Thailand; chaung_17@hotmail.com

**Keywords:** carapace, circadian rhythm, digestive enzyme, feces, head-starting program, health, meal frequency, meal interval

## Abstract

**Simple Summary:**

Head-starting programs of hatchlings before release of yearlings to natural habitat are an alternative approach for restoring the population of critically endangered sea turtles. Hawksbill sea turtle has been reared in captivity programs in several countries, while the feeding regimens have never been optimized. In the current study, the feeding frequency of hawksbill sea turtle was optimized in indoor experimental conditions. Two-month-old turtles were fed at different frequencies: one meal daily at 12.00 h, two meals daily at 08.00 and 12.00 h, two meals daily at 08.00 and 16.00 h, two meals daily at 12.00 and 16.00 h or three meals daily at 08.00, 12.00, and 16.00 h. At the end of an 8-week trial, two meals daily with long time interval (at 08.00 and 16.00 h) were optimal, based on assessment criteria of growth, feed utilization, digestive enzyme markers, available and unavailable nutrients present in the feces, hematological parameters, and carapace elemental composition. This finding could be directly used as a feeding guideline supporting the head-starting programs of this species.

**Abstract:**

Hawksbill sea turtle (*Eretmochelys imbricata*) has been reared in head-starting captivity programs, while the feeding regimens have never been optimized. In the current study, the feeding frequency of hawksbill sea turtle was investigated in indoor experimental conditions. Two-month-old turtles (38.98 ± 0.02 g) were distributed to triplicates of five treatments containing three turtles each and they were fed at different frequencies: one meal daily at 12.00 h (1M12), two meals daily at 08.00 and 12.00 h (2M8–12), two meals daily at 08.00 and 16.00 h (2M8–16), two meals daily at 12.00 and 16.00 h (2M12–16), or three meals daily at 08.00, 12.00 and 16.00 h (3M8–12–16). At the end of an 8-week trial, growth performance (specific growth rate 2.39 ± 0.02% body weight day^−1^) and feed consumption (feeding rate 2.00 ± 0.43 g day^−1^) were highest for turtles fed 2M8–16, followed by 2M12–16 or 3M8–12–16 relative to the other treatments (*p* ˂ 0.05). These treatments had significantly higher trypsin specific activity and trypsin/chymotrypsin ratio, and *vice versa* for lipase specific activity and amylase/trypsin ratio, relative to the remaining treatments. These match well with the fecal thermal properties that indicate amounts of available and unavailable nutrients present in the feces. Hematological parameters and carapace elemental composition showed no negative effects to turtles in 2M8–16 treatment. Therefore, two meals daily with long time interval were optimal for feeding hawksbill sea turtle. Findings from the current study could be directly used as a feeding guideline supporting the head-starting programs of this species.

## 1. Introduction

Hawksbill sea turtle (*Eretmochelys imbricata*) is listed as a critically endangered marine species by the International Union for Conservation of Nature, the Convention on International Trade in Endangered Species of Wild Fauna and Flora (Appendix I), the Convention on Migratory Species, and also the Wild Animal Reservation and Protection Act of Thailand (B.E. 2562). This species distributes throughout tropical and subtropical areas of the Atlantic, Indian, and Pacific Oceans, and nests in some 60 of the 108 countries they inhabit [1]. The population of hawksbill sea turtles has continued to decline due to shell trade, egg collection, slaughter for meat, destruction of nesting and foraging habitats, hybridization with other species, oil contamination, and ingestion of marine debris [2]. In some countries, such as in Thailand, head-starting captivity programs of hatchlings to yearlings before release to natural habitats have been conducted. At the end of such program, it is believed that the size and the carapace strength of head-started turtles are sufficient to escape from some predators, such as shorebirds, shore crabs, large fish, sharks, ants, snakes, goannas, and mammals, increasing the chances of survival until adulthood in the natural habitats [3,4].

Under captive programs, some feeding regimens for sea turtles are partially available. However, since these are not commercially important cultivated species, all relevant parameters have not yet been optimized. In natural habitats, hawksbill sea turtle feeds on seaweeds, seagrasses, sea sponges, cnidarians, mollusks, crustaceans, ascidians, and small fish [5,6]. In Thailand, floating pellet feed for marine carnivorous fish is used during the head-starting programs of this species, whereas only little is known about the management of feeding regimens.

Feeding captive animals with dry pellets can cause floating-bloating problems due to overfeeding [7] and is also known to induce obesity [8]. In the various feeding regimens, feeding frequency is an important parameter contributing to growth and successful feed utilization. Across turtle species, optimal feeding frequency of two meals daily has been reported in green turtle, *Chelonia mydas* [9] and soft-shelled turtle, *Pelodiscus sinensis* [10], while this optimization has not yet been conducted in the other species. On the other hand, various reports have addressed well-known fish species cultivated for commercial purposes [11,12,13,14].

Feeding frequencies or feeding intervals have a direct effect on feed utilization [9], affecting post-prandial secretion and retention time of digestive enzymes responding to food intake [15]. Therefore, digestive enzymes along the alimentary tract can be used to monitor feed utilization in such an experiment. However, for endangered species the digestive enzymes extracted from feces can also trace this physiological response. Not only does this show a similar pattern with the enzymes from digestive tract [16], but also the sampling of feces does not intrude on the captive reared animals. In addition, as a non-invasive technique, the thermal property study can also be used to detect unavailable and available nutrients in feces after digestion and absorption. This technique can be applied in the feeding trials of various aquatic animal models [9,17,18,19].

The well-being of turtles in a captivity program can be assessed from the carapace elemental composition and hematological determinations [9,17,18]. This is because the feeding regimens directly influence the metabolites present in blood and the accumulation of nutrients in the carapace. Both observables are significantly influenced by feeding regimens, according to previous reports, such as feeding frequency [9] and pre-soaking ratio of feed pellet in water before use for feeding [18]. Therefore, the optimal feeding frequency in the current study was investigated based on growth and feed utilization, fecal digestive enzyme activities, fecal thermal properties, carapace elemental composition, and hematological parameters. The findings from current study could be directly applied in pond or aquarium management for head-starting this endangered species.

## 2. Materials and Methods

### 2.1. Animal Ethics

This project was conducted at Marine Endangered Species Unit, Phuket Marine Biological Center, Phuket, Thailand, and followed the Wild Animal Reservation and Protection Act of Thailand (B.E. 2562). The preparation, husbandry, feeding trial and sampling of animals conformed to the “Ethical Principles and Guidelines for the Use of Animals for Scientific Purposes”, National Research Council, Thailand (Application No. U1-06514-2560).

### 2.2. Preparation and Husbandry of Hatchlings

One hundred and thirty-one eggs were collected from a natural spawning hole of hawksbill sea turtle at Khram Island, Chon Buri, Thailand, under conservation programs of the Department of Marine and Coastal Resources. All eggs were transported to an artificial sand pit and left to incubate for 50 days. One hundred and twenty-six hatchlings emerged and survived (96.18% survival). They were starved for 15 days until reaching the end of yolk sac stage. The turtles were reared in cement ponds (1 m × 2 m) with 10 cm water depth and were fed to satiation once per day (08.00 h) with yellow-stripe scad fillet (*Selaroides leptolepis*). The 45-day-old turtles were transported to Marine Endangered Species Unit, Phuket Marine Biological Center, for acclimatization. All the turtles were fed to satiation two meals daily (08.00 and 16.00 h) by commercial pellets (5 mm diameter of pellet size) for marine carnivorous fish (Charoen Pokphand PCL., Samut Sakhon, Thailand). The proximate chemical compositions contained 48.80% crude protein, 6.98% crude lipid, 1.77% crude fiber, 12.10% crude ash, 30.35% nitrogen-free extract, and 3794 kcal kg^−1^ energy on dry matter basis. The main ingredients in feed included fish meal, squid liver meal, soybean meal, wheat flour, wheat bran, fish oil, and vitamin–mineral premix.

### 2.3. Feeding Test

Two-month-old turtles with similar weights (38.98 ± 0.02 g initial body weight, *n* = 45) were distributed to triplicates of five treatments containing three turtles each. They were fed *ad libitum* at different frequencies: one meal daily at 12.00 h (1M12), two meals daily at 08.00 and 12.00 h (2M8–12), two meals daily at 08.00 and 16.00 h (2M8–16), two meals daily at 12.00 and 16.00 h (2M12–16), or three meals daily at 08.00, 12.00, and 16.00 h (3M8–12–16), using the commercial pellets for marine carnivorous fish described above. All the experimental units were hosted in plastic tanks (54 cm diameter × 21 cm depth, with a 10 cm water level) under 12 h light (06.00 to 18.00 h): 12 h dark natural cycle for the 8-week experimental period. Water was entirely changed daily at 07.30 h, and its quality during the study was: pH 8.16 ± 0.07, temperature 28.92 ± 0.10 °C, and salinity 32.00 ± 0.19 g L^−1^. Mortality and morbidity were monitored and recorded daily. Growth performance, in terms of body weight (BW), straight carapace width, and straight carapace length was recorded every other week over the duration of the experiment. These values were used for calculating weight gain, specific growth rate (SGR), straight carapace width gain, straight carapace length gain, and body condition index (BCI). The uneaten feed was immediately collected by dip-net 30 min after feeding. Feeding rate and feed conversion ratio (FCR) were calculated from the weight differences between feed provided and uneaten feed, after oven-drying at 60 °C until constant weight. All the growth parameters and the samples for further analyses (carapace and blood) were collected after fasting the turtles for 24 h. Calculation of the parameters followed the equations below:

Survival (%) = (Final turtle number/initial turtle number) × 100(1)

Weight gain (g) = Final BW (g) − initial BW (g)(2)

Straight carapace width gain (cm) = Final straight carapace width (cm) − initial straight carapace width (cm)(3)

Straight carapace length gain (cm) = Final straight carapace length (cm) − initial straight carapace length (cm)(4)

SGR (% BW day^−1^) = [(ln W_t_ − ln W_o_)/(t − t_o_)] × 100 (5)

where W_t_ = mean BW (g) at day t, W_o_ = mean BW (g) at day t_o_.

BCI (kg cm^−3^) = [BW (kg)/final straight carapace length (cm)^3^] × 10^4^ (6)

FCR (g feed g gain^−1^) = Dry feed consumed (g)/wet weight gain (g) (7)

### 2.4. Determination of Fecal Digestive Enzymes

At the end of 8-week feeding trial, turtle feces (*n* = 3 per treatment) were quickly collected by dip-net after removing the uneaten feed of the last meal. One sample was collected from each tank until obtain desired weight for enzyme extraction (0.5 g) and then they were carefully rinsed with cold distilled water three times. The samples were mixed with cold distilled water at a ratio of 1:3 *w*/*v* and then were homogenized using a micro-homogenizer (THP-220; Omni International, Kennesaw, GA, USA). Centrifugation was performed at 15,000× *g* for 30 min at 4 °C to obtain supernatants. The 200 µL aliquots were kept in microcentrifuge tubes at –20 °C until use. The protein concentration in a crude extract was determined according to the standard method of Lowry et al. [20], using bovine serum albumin as protein standard. All assays of digestive enzymes and protein concentration were performed within one month after extraction.

The optimal conditions for fecal digestive enzyme assays were chosen from the previous report on green turtles [21]. Hemoglobin was used as non-specific substrate for determination of pepsin activity (EC 3.4.23.1), according to the method of Worthington [22]. The liberated soluble peptides were spectrophotometrically measured at 280 nm, and a 1.0 increase in absorbance was defined as one unit (U) of enzyme. *N*-benzoyl-*L*-Arg-*p*-nitroanilide (BAPNA) and *N*-succinyl-Ala-Ala-Pro-Phe-*p*-nitroanilide (SAPNA) were used as specific substrates for respective trypsin (EC 3.4.21.4) and chymotrypsin (EC 3.4.21.1) assays, according to Rungruangsak-Torrissen et al. [23]. The same liberated product for both enzymes was measured at 410 nm and compared with linear range of *p*-nitroanilide standard curve. Soluble starch was used as substrate for amylase activity assay (EC 3.2.1.1) based on Bernfeld [24]. The liberated product was quantified at 540 nm against linear range of maltose standard curve. The *p*-nitrophenyl palmitate was used as substrate for lipase activity assay (EC 3.1.1.3) based on Winkler and Stuckmann [25]. The product was spectrophotometrically measured at 410 nm against *p*-nitrophenol standard. All liberated products from digestive enzyme assay were measured using a 6-position cell holder spectrophotometer (Genesys 10S UV–Vis, Thermo Fisher Scientific, Madison, WI, USA). One unit of trypsin, chymotrypsin, amylase, and lipase is defined as the amount that catalyzes the conversion of 1 μmol of substrate per min. The activity ratios, amylase to trypsin (A/T ratio), and trypsin to chymotrypsin (T/C ratio), were calculated using the enzyme activities for the same samples.

### 2.5. Thermal Properties of Feces

The same fecal samples (*n* = 3 per treatment), as previously described in digestive enzyme section, were dried using a freeze dryer (Delta 2-24 LSC; Martin Christ Gefriertrocknungsanlagen GmbH, Osterode am Harz, Germany) for 24 h. The dried samples were mashed to obtain homogenous powder using mortar and pestle. The thermal response of three milligrams dried feces was run from 40 to 400 °C at a rate of 10 °C min^−1^ against an empty reference pan. Thermograms and thermal properties, in terms of onset (T_o_), peak (T_p_), and conclusion (T_c_) temperatures and enthalpy (∆H), were recorded using a differential scanning calorimeter (DSC7; Perkin Elmer, Waltham, MA, USA).

### 2.6. Elemental Composition in Carapace

The carapaces (1 mm × 1 mm, *n* = 3 per treatment) from supracaudal scute were dissected by aseptic scissors; this amputation area can regenerate within one month after dissection [9]. The samples were dried using a hot air oven at 60 °C for 24 h to remove the moisture. The elemental composition was semi-quantitatively determined using a scanning electron microscope (Quanta 400; FEI, Brno, Czech Republic) equipped with an energy dispersive X-ray spectrometer (X-MAX; Oxford Instruments, Oxford, UK). The accelerating voltage was set at 20 kV, and high vacuum mode and silicon drift detector were used. Ten random points of each carapace sample were analyzed for elemental composition.

### 2.7. Hematological Parameters

The blood samples (*n* = 3 per treatment) were collected from dorsal cervical sinus. All hematological parameters were determined within 12 h after collection. Blood suspension was prepared [26] and red and white blood cells were counted with a hemacytometer (Precicolor; HBG, Giessen-Luetzellinden, Germany) under a compound microscope. The hemoglobin was determined by measuring the formation of cyanmethemoglobin according to the method of Larsen and Snieszko [27]. The hematocrit was determined by using laboratory-prepared capillary tubes treated with 10% heparin according to the method of Larsen and Snieszko [28]. The cholesterol concentration was determined via the activities of cholesterol oxidase and peroxidase [29], while triglycerides were determined, using a mixture of lipoprotein lipase, glycerol kinase, glycerol phosphate oxidase, and peroxidase [30]. The respective products of both enzymatic methods were spectrophotometrically measured (Genesys 10S UV–Vis, Thermo Fisher Scientific, Madison, WI, USA) at 500 and 505 nm. Total protein was quantified using Folin phenol reagent, and colorimetric changes were spectrophotometrically measured at 750 nm against linear range of bovine serum albumin [20]. Blood urea nitrogen, creatinine, alkaline phosphatase, alanine transaminase and aspartate aminotransferase were determined using a commercial diagnostic kit (PZ Cormay S.A. Company, Lomianki, Poland).

### 2.8. Statistical Analysis

Statistical analyses were performed with Statistical Package for Social Science Software Version 14 (SPSS Inc., Chicago, IL, USA). Arc sine transformation was applied to percentages prior to analysis. One-Way ANOVA was used, and the mean *post hoc* comparisons were carried out using Duncan’s multiple range test at significance level α = 0.05 (*p* ˂ 0.05). The relationships for each pair of digestive enzymes were evaluated using Pearson correlations, expressed by the correlation coefficients (*r*).

## 3. Results

### 3.1. Survival, Growth Performance and Feed Utilization

No mortality was observed during the eight weeks of experiment. Growth performance (final body weight, weight gain and SGR) was highest in turtles fed 2M8–16, followed by 2M12–16 or 3M8–12–16, 2M8–12, and 1M12 in this rank order (*p* < 0.05). The highest final straight carapace length and straight carapace length gain obtained in turtles fed 2M8–16 or 3M8–12–16, followed by 2M8–12 and 2M12–16 treatments, while the straight carapace width gain was highest in turtles fed 2M8–16, followed by the other remaining treatments receiving two or three meals daily. The final straight carapace width and BCI did not differ across the dietary treatments. Feeding rate was significantly higher in 2M8–16 and 3M8–12–16 treatments than in 1M12 and 2M8–12 groups, while FCR showed no significant differences across the dietary treatment groups (Table 1).

### 3.2. Fecal Digestive Enzyme Activity

**Figure 1:**
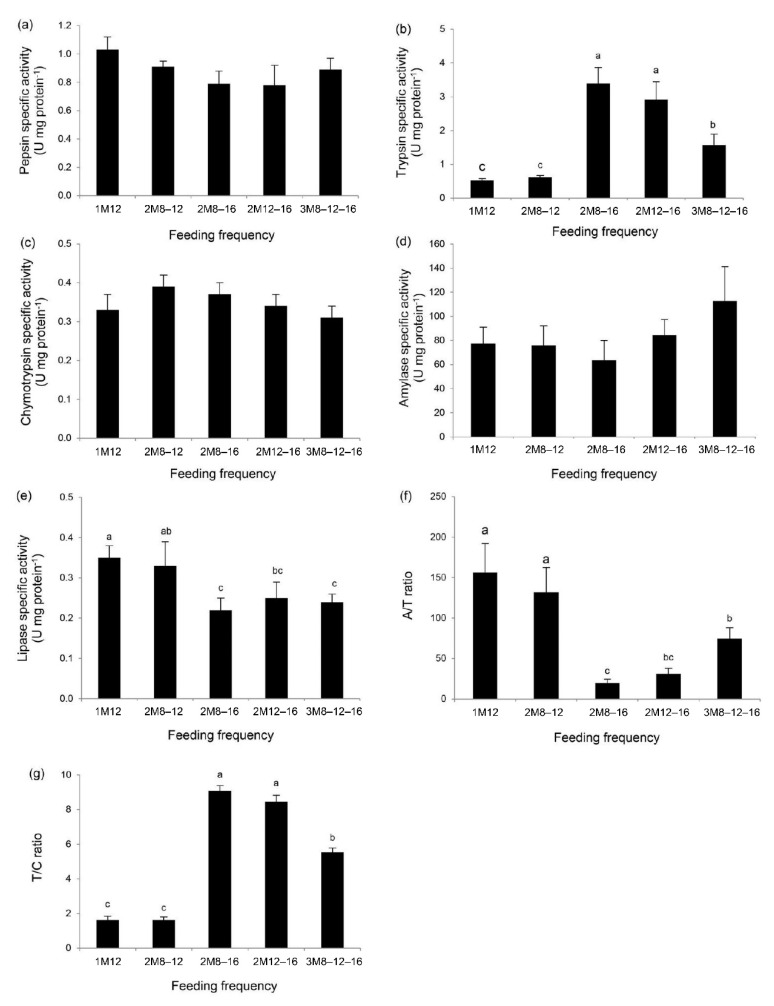
Specific activities of fecal digestive enzymes in hawksbill sea turtle fed at different frequencies: pepsin (**a**), trypsin (**b**), chymotrypsin (**c**), amylase (**d**), lipase (**e**), A/T ratio (**f**), and T/C ratio (**g**). The feces were sampled at the end of the eight-week experiment. Data are expressed as tank mean ± SEM. Different superscripts indicate a significant difference (*p* < 0.05). A/T ratio is the ratio of amylase to trypsin; T/C ratio is the ratio of trypsin to chymotrypsin.

No differences in the specific activity of pepsin were found due to varying the feeding frequency (Figure 1a). Trypsin specific activity was the highest in turtles fed 2M8–16 and 2M12–16, followed by 3M8–12–16 treatment (Figure 1b). Similar response as in pepsin was observed in specific activities of chymotrypsin (Figure 1c) and amylase (Figure 1d). Lipase specific activity was the highest in turtles fed 1M12 or 2M8–12, followed by 2M12–16 (Figure 1e). Similar change was also observed in A/T ratio (Figure 1f). The T/C ratio responded to the feeding frequency similarly as trypsin (Figure 1g).

Pearson correlation analysis indicated significant relationships between pairs of fecal digestive enzymes across the five groups with varied feeding frequency. Pepsin exhibited a positive relationship with trypsin, chymotrypsin, and T/C ratio, but was negatively correlated with lipase and A/T ratio. Trypsin positively correlated with chymotrypsin and T/C ratio, and *vice versa* for its reciprocal and lipase. The relationships between chymotrypsin with A/T ratio or lipase, and T/C ratio with A/T ratio, were negatively correlated. Lipase showed significant negative relationship with T/C ratio but was positively correlated with A/T ratio. No significant relationship was observed for amylase with any other enzyme (Table 2).

### 3.3. Fecal Thermal Properties

Four peaks were observed in DSC thermograms across all the five dietary treatments. The two low temperature peaks were in the range from 44.07 to 106.38 °C, while the high temperature peaks were in the range from 130 to 360.58 °C. Generally, thermal properties in terms of T_o_, T_p_, T_c_, T_c_–T_o_ and ΔH were different between the dietary treatment groups. Turtles fed 2M12–16 exhibited the highest ΔH_1+2_, ΔH_3+4_, and ΣΔH, followed by 2M8–16, while the lowest enthalpic response was noted with the 1M12 treatment (Table 3).

### 3.4. Carapace Elemental Composition

Six major elements were observed in the carapace samples of hawksbill sea turtles, but all of these (carbon, nitrogen, oxygen, sulfur, sodium, and chlorine) showed no differences between the treatments (Table 4).

### 3.5. Hematological Parameters

Feeding frequency had no significant effects on hematological parameters observed in the current study, including red blood cells, hemoglobin, hematocrit, white blood cells, blood urea nitrogen, cholesterol, triglyceride, high-density lipoprotein-cholesterol, low-density lipoprotein-cholesterol, total protein concentration, blood performance, aspartate aminotransferase, alanine transaminase, and alkaline phosphatase (Table 5).

## 4. Discussion

### 4.1. Growth and Feed Utilization of Reared Turtles

Hawksbill sea turtles fed by 2M8–16 had a better performance, followed by 2M12–16 or 3M8–12–16. The optimal frequency found from the current study matches with the routine feeding schedule of hawksbill sea turtles in Thailand, as well as with the reported head-starting programs of the same species [32] and northern river terrapins, *Batagur baska* [17]. Moreover, two meals daily (07.00 and 16.30 h) is appropriate for green turtles when compared with one (13.30 h), three (07.00, 11.30, and 16.30 h) and four meals daily (07.00, 10.30, 13.30, and 16.30 h) [9], or for soft-shelled turtles when comparing two meals daily (10.00 and 17.00 h) with one meal daily (17.00 h) [10]. In hatchlings and yearlings of Kemp’s ridley sea turtles (*Lepidochelys kempii*), feeding two meals daily (early morning and late afternoon) at respective rations from 5.0% to 1.5% of body weight per day has been used in captivity programs [7]. In addition, this preferred frequency has been reported for some fish species, such as yellowtail flounder, *Limanda ferruginea* [33]; and mono-sex male Nile tilapia, *Oreochromis niloticus* [14].

Furthermore, meal intervals and circadian rhythm directly affected growth performance and feed utilization in the current study, since there were differences in growth parameters between 2M8–12, 2M12–16, and 2M8–16. Physiologically, feeding too often (at short intervals) can cause food to pass through the digestive tract quickly, resulting in less effective digestion [34]. Therefore, the meal interval in the optimal frequency might improve digestion and absorption along the alimentary tract. In loggerhead sea turtles (*Caretta caretta*), the food transit time and the retention time are around 9 and 12 h after feeding [35]. While these aspects have not yet been investigated in hawksbill sea turtles, this range is close with the time interval 8 h (2M8–16) in the current study, relative to the 4 h in 2M8–12 and 2M12–16. It is reasonable that feeding two meals daily with 8 h interval should give a growth increment and effective feed utilization. For circadian rhythm, improved performance was observed in turtles fed 2M12–16 relative to 2M8–12. This might be due to the activity of turtles during daytime, so that high energy consumption, resulting decreased growth and feed utilization, were all observed in 2M8–12 group of the turtles. A circadian clock mechanism has been reported to affect swimming activity of hatchling hawksbill sea turtle [36]. In addition, circadian or circannual rhythms have been reported in some turtles, such as red-eared slider, *Trachemys scripta elegans* [37]. Therefore, these issues may be raised for further improving feeding regimens in this species. The connection between blood biochemistry and response of animals to feeding might provide insights into the physiological response of turtles due to feeding frequency, meal intervals, and circadian rhythm.

Reduced feeding rate indicates that the received nutrients are not sufficient to maintain growth and development in the turtles fed 1M12 and 2M8–12. Therefore, the animals will ingest a large amount of food per meal [38]. On the other hand, high feeding rate within short meal interval (2M12–16 and 3M8–12–16) might result in increased amount of energy used for excretion and increased production of nitrogen waste; feeding rate does not proportionally relate to growth rate. In the current study, the turtles can compensate FCR over the range from one to three meals per day. It is possible that the complicated interactions of feeding rate, growth response and energy used for excretion of excessive nutrients can lead to unchanged FCR. The values of FCR from 0.80 to 1.02 are relatively low, which is a good trait. Similar range of FCR has been reported in green turtle [39] and in northern river terrapin [40], having respective ranges from 0.61 to 0.81, and from 1.03 to 1.46. Here the estimation of consumed pellet feed was based on dry matter, while the weight gain of turtles includes a large fraction of water in the tissues because it is on wet weight basis.

### 4.2. Fecal Digestive Enzyme Specific Activities in Reared Turtles

Feed utilization can be assessed through the activity of digestive enzymes. In endangered species, the digestive enzymes from feces can trace physiological response due to varying dietary treatments [9,18,39]. A similar pattern of trypsin from feces and digestive tract has been reported in crustacean [16] while the comparison between enzyme sources in reptiles has not been performed. Since non-invasive sampling is essential for endangered species, comparison in activity outputs of digestive enzymes between feces and intestine needs to be assessed before using as nutritional and physiological indicators. In the current study, across the three types of proteolytic enzymes observed, only trypsin was significantly affected. This enzyme cleaves the peptide chain on the C-terminal end of arginine and lysine residues, while pepsin and chymotrypsin preferably cleave the peptides in different positions [41]. Generally, trypsin contributes 40–50% of the protein digestion in alimentary tract of fish [42]. In various fish species, increased trypsin and T/C ratio are associated with feeding conditions favoring growth and feed utilization [23,43]. Significantly increased trypsin specific activity and T/C ratio in the current study indicate high capacity of turtles to grow and improve feed utilization. These data match well with the correlation coefficients between trypsin specific activity and SGR (*r* = 0.862, *p* < 0.01, *n* = 15) or feeding rate (*r* = 0.758, *p* < 0.01, *n* = 15), and between T/C ratio and SGR (*r* = 0.903, *p* < 0.01, *n* = 15) or feeding rate (*r* = 0.750, *p* < 0.01, *n* = 15).

Amylase cleaves glycosidic links in starch, producing maltose and maltotriose from amylose as well as glucose, maltose, and dextrin from amylopectin; this enzyme plays important roles in utilizing carbohydrates in the forms of nitrogen-free extract. In the current study, amylase activity was unchanged across a wide range of feeding regimens. This is because the liberated glucose serves as the sole source of energy for a number of tissues [44]. A similar finding was also observed in green turtles subjected to feeding frequencies of one to four meals daily [9]. However, significantly decreased A/T ratio, a marker for assessing the use of carbohydrates per amount of proteins [45,46], was observed in turtles fed 2M8–16, 2M12–16 and 3M8–12–16. This indicates that turtles can mainly utilize protein (mainly digested by trypsin) under these feeding regimens. It is possible that the juvenile turtles in these three groups can physiologically maintain carnivorous feeding habit as observed in nature, while feeding 1M12 and 2M8–12 might induce the turtles to be omnivorous, which is dominantly plant-eating.

Lipase cleaves carboxylic ester bonds between glycerol and fatty acids. Therefore, it performs essential roles in digestion, transport, and processing of dietary lipids in food. In the current study, relatively low lipase specific activity was observed in turtles fed 2M8–16, 2M12–16, and 3M8–12–16, concurrently providing negative correlation with specific activities of protein-digesting enzymes (pepsin, trypsin, and chymotrypsin). This opposite direction of enzymatic activities might play a key role in controlling nutrient utilization under this feeding regimen. It is possible that protein-sparing effect occurs by increasing lipase specific activity, as well as A/T ratio, when the activities of proteolytic enzymes decreased. This means that once turtles were fed two meals daily (2M8–16 and 2M12–16) or 3M8–12–16, their bodies essentially will use protein for growth increment; while a turtle with 1M12 or 2M8–12 will have to eat whatever is available and probably uses lipids as a sole source due to limitation of protein catabolism. The responses in 1M12 and 2M8–12 treatments are in agreement with the observation in sea turtles by Moon et al. [47] who indicate that lipids and glycogen may primarily be used as energy during short-term fasting, and probably with short-term intervals between meals in the current study. If an artificial feed is developed for this species, fat-enriched high-energy feed may result in rapid growth and favorable feed conversion [48].

### 4.3. Nutrients Present in Feces and Parameters Indicating Health

Significant changes in digestive enzyme activities relate with fecal thermal properties, since the thermogram from DSC reflects the native amount of nutrients present in feces after digestion and absorption [9,18,21,49]. Generally, the low temperature peaks (peaks 1 and 2) detect available nutrients, mainly protein, nitrogen-free extract, and lipid, while the high temperature peaks (peaks 3 and 4) refer to unavailable composition, probably crude fiber [40]. The T_o_, T_p_, T_c_, T_c_–T_o_, and ΔH were different between groups in the current study, indicating the effects of feeding regimen on feed utilization efficiency. Significantly decreased ΔH_1+2_, together with the lowest feeding rate, indicate insufficient nutrients for turtles fed 1M12. Similar phenomenon is also observed in turtles fed 2M8–12. On the other hand, the turtles fed 2M12–16 appear to have received a higher amount of feed than what they expended in activities, so that high residual nutrients were detected in the feces. For the remaining treatments, the thermal responses showed proper characteristics but the complicated interactions between feed consumption and fecal digestive enzyme activity would raise the growth performance of 2M8–16 over 3M8–12–16 groups. It is possible that turtles fed at the highest frequency can ingest less feed per meal; this might help the enzymes to access nutrients, leading to a lesser amount of nutrients detected in feces. However, it should be noted that increase of digestive enzyme activities does not mean improved growth all times as producing digestive enzyme consume energy as well. Further, it still is not clear whether increased digestive enzymes caused improved growth rate or *vice versa*. In other words, it is not clear which one is cause and which one is the effect.

The well-being issue is widely raised for animals reared in captive programs. No differences in BCI indicate normal morphometric changes of reared turtles over the range of feeding frequencies tested. Generally, elemental composition in carapace reflects the varying feeding regimens or the physical conditions where the animals lived [9,17,18,40], whereas hematological parameters are influenced by seasonal changes, age, sex, geographical location, physiological responses, and reproductive status [50]. No differences in carapace elemental composition and hematological parameters between the turtles fed 2M8–16 and the other remaining treatments suggest no negative effects on health status by the treatments.

## 5. Conclusions

Receiving from one to three meals daily in varying feeding regimens, juvenile hawksbill sea turtles exhibited superior traits of growth and feed utilization when fed by the commercial feed two meals daily at 8 h daytime interval (08.00 and 16.00 h). This frequency had no negative effects on the well-being or welfare, as indicated by carapace elemental composition or hematological parameters. However, lack of any change in carapace elemental composition or hematological parameters can be due to too few numbers of turtles per tank. Fighting with each other and have social challenges and feeding hierarchies that all issues cause stress and, therefore, changes in these parameters. Findings from the current study can serve as practical guidelines for feeding management in head-starting programs of juvenile hawksbill sea turtles. In addition, the findings from the current study also pointed to the effects of meal intervals in relation to circadian rhythms; these issues should be of interest in improving hawksbill sea turtle husbandry programs.

## Figures and Tables

**Table 1:** Survival, growth performance, and feed utilization of hawksbill sea turtles fed at various frequencies. The observed parameters were recorded at the end of the 8-week experiment.

Parameter	1M12	2M8–12	2M8–16	2M12–16	3M8–12–16	*p*-Value
Survival (%)	100	100	100	100	100	–
FBW (g)	107.71 ± 0.64 ^d^	114.92 ± 1.87 ^c^	148.81 ± 1.46 ^a^	139.11 ± 2.51 ^b^	138.48 ± 0.91 ^b^	< 0.001
WG (g)	68.73 ± 0.62 ^d^	75.93 ± 1.06 ^c^	109.83 ± 0.94 ^a^	100.17 ± 1.21 ^b^	99.49 ± 0.74 ^b^	< 0.001
SGR (% BW day^−1^)	1.82 ± 0.01 ^d^	1.93 ± 0.03 ^c^	2.39 ± 0.02 ^a^	2.27 ± 0.03 ^b^	2.26 ± 0.01 ^b^	< 0.001
FSCW (cm)	6.89 ± 0.33	7.38 ± 0.70	8.22 ± 0.03	7.37 ± 0.49	7.43 ± 0.09	0.065
SCWG (cm)	1.75 ± 0.10 ^c^	2.19 ± 0.02 ^b^	3.08 ± 0.07 ^a^	2.15 ± 0.17 ^b^	2.20 ± 0.08 ^b^	0.036
FSCL (cm)	8.27 ± 0.33 ^c^	8.96 ± 0.12 ^b^	9.72 ± 0.07 ^a^	9.06 ± 0.17 ^b^	9.67 ± 0.08 ^a^	0.001
SCLG (cm)	2.09 ± 0.31 ^c^	2.76 ± 0.10 ^b^	3.56 ± 0.14 ^a^	2.87 ± 0.19 ^b^	3.42 ± 0.12 ^a^	0.002
BCI (kg cm^−3^)	1.94 ± 0.24	1.60 ± 0.07	1.62 ± 0.02	1.88 ± 0.12	1.53 ± 0.02	0.148
FR (g day^−1^)	0.98 ± 0.05 ^c^	1.27 ± 0.09 ^bc^	2.00 ± 0.43 ^a^	1.64 ± 0.12 ^ab^	1.75 ± 0.15 ^a^	0.004
FCR (g feed g gain^−1^)	0.80 ± 0.06	0.94 ± 0.11	1.02 ± 0.37	0.92 ± 0.10	0.98 ± 0.11	0.327

1M12, one meal daily at 12.00 h; 2M8–12, two meals daily at 08.00 and 12.00 h; 2M8–16, two meals daily at 08.00 and 16.00 h; 2M12–16, two meals daily at 12.00 and 16.00 h; 3M8–12–16, three meals daily at 08.00, 12.00 and 16.00 h; FBW, final body weight; WG, weight gain; SGR, specific growth rate; BW, body weight; FSCW, final straight carapace width; SCWG, straight carapace width gain; FSCL, final straight carapace length; SCLG, straight carapace length gain; BCI, body condition index; FR, feeding rate; FCR, feed conversion ratio; Data are expressed as tank mean ± SEM; Differences between means were tested with Duncan’s multiple range test; Different superscripts in the same row indicate a significant difference (*p* < 0.05).

**Table 2:** Correlation coefficients (*r*) between each pair of fecal digestive enzymes. The values were calculated from experimental units across all feeding frequencies observed (*n* = 15).

Digestive Enzyme	Pepsin	Trypsin	Chymotrypsin	Lipase	Amylase	A/T ratio	T/C Ratio
Pepsin	1						
Trypsin	0.632 *	1					
Chymotrypsin	0.968 **	0.651 **	1				
Lipase	–0.630 *	–0.690 **	–0.730 **	1			
Amylase	0.223	–0.097	0.157	–0.038	1		
A/T ratio	–0.528 *	–0.707 **	–0.587	0.607 *	0.457	1	
T/C ratio	0.737 **	0.967 **	0.737 **	–0.710 **	0.041	–0.712	1

A/T ratio is activity ratio of amylase to trypsin; T/C ratio is activity ratio of trypsin to chymotrypsin; *, ** The relationships between each pair were significantly correlated at *p* < 0.05 and *p* < 0.01, respectively.

**Table 3:** Thermal properties for detecting available and unavailable nutrients present in feces of hawksbill sea turtle subjected to feeding at various frequencies. The sampling was done at the end of the 8-week experiment.

Thermal Property	Peak	T_o_ (°C)	T_p_ (°C)	T_c_ (°C)	T_c_–T_o_ (°C)	ΔH (J g^−1^)	ΔH_1+2_ (J g^−1^)	ΔH_3+4_ (J g^−1^)	ΣΔH (J g^−1^)
1M12	1	44.07	63.33	79.42	27.09	31.28	35.67	16.38	52.05
	2	91.15	97.89	105.13	13.98	4.39			
	3	130.00	138.17	149.86	19.86	2.57			
	4	313.07	331.39	347.40	34.33	13.81			
2M8–12	1	46.61	64.33	79.90	33.29	53.67	56.49	25.18	81.67
	2	92.62	98.06	104.04	11.42	2.82			
	3	137.58	144.00	153.75	16.17	4.40			
	4	306.14	325.61	355.92	49.78	20.78			
2M8–16	1	44.25	60.39	77.51	33.26	51.10	58.11	44.21	102.32
	2	89.91	96.44	103.45	13.54	7.01			
	3	133.18	138.17	144.04	3.68	10.86			
	4	311.29	338.83	348.91	37.62	33.35			
2M12–16	1	45.29	65.00	82.78	37.49	153.11	165.15	88.08	253.23
	2	92.52	100.00	106.38	13.86	12.04			
	3	135.38	146.17	158.64	23.27	8.89			
	4	315.47	336.17	360.58	45.11	79.19			
3M8–12–16	1	44.66	63.28	79.83	35.17	39.61	45.28	39.58	84.86
	2	91.61	99.33	105.02	13.41	5.67			
	3	136.56	145.08	160.66	24.10	5.75			
	4	302.81	315.56	327.58	20.25	33.83			

1M12, one meal daily at 12.00 h; 2M8–12, two meals daily at 08.00 and 12.00 h; 2M8–16, two meals daily at 08.00 and 16.00 h; 2M12–16, two meals daily at 12.00 and 16.00 h; 3M8–12–16, three meals daily at 08.00, 12.00, and 16.00 h; T_o_, onset temperature; T_p_, peak temperature; T_c_, conclusion temperature; T_c_–T_o_, melting temperature range; ΔH, transition enthalpy; Data are expressed as means from triplicates.

**Table 4:** Elemental composition (% of dry weight) in carapace of hawksbill sea turtle subjected to feeding at various frequencies. The sampling was done at the end of the 8-week experiment.

Element	1M12	2M8–12	2M8–16	2M12–16	3M8–12–16	*p*-Value
Carbon	51.36 ± 1.19	52.11 ± 0.89	50.30 ± 0.59	50.92 ± 0.31	48.71 ± 0.52	0.084
Oxygen	27.92 ± 0.66	26.78 ± 0.66	28.57 ± 0.70	27.64 ± 0.29	29.33 ± 0.72	0.125
Nitrogen	19.75 ± 0.67	20.19 ± 0.37	20.10 ± 0.12	20.54 ± 10.26	21.31 ± 0.30	0.122
Sulfur	0.69 ± 0.17	0.77 ± 0.11	0.60 ± 0.04	0.55 ± 0.06	0.44 ± 0.05	0.274
Sodium	0.24 ± 0.01	0.19 ± 0.01	0.25 ± 0.02	0.24 ± 0.08	0.25 ± 0.03	0.753
Chlorine	0.20 ± 0.01	0.17 ± 0.02	0.22 ± 0.04	0.14 ± 0.01	0.14 ± 0.01	0.109

1M12, one meal daily at 12.00 h; 2M8–12, two meals daily at 08.00 and 12.00 h; 2M8–16, two meals daily at 08.00 and 16.00 h; 2M12–16, two meals daily at 12.00 and 16.00 h; 3M8–12–16, three meals daily at 08.00, 12.00, and 16.00 h; Data are expressed as tank mean ± SEM; Differences between means were tested with Duncan’s multiple range test; Different superscripts in the same row indicate a significant difference (*p* < 0.05).

**Table 5:** Hematological parameters of hawksbill sea turtle subjected to feeding at various frequencies. The sampling was done at the end of the 8-week experiment.

Hematological Parameter	1M12	2M8–12	2M8–16	2M12–16	3M8–12–16	*p*-Value
RBC (×10^5^ cells µL^−1^)	4.80 ± 0.91	3.93 ± 0.88	3.70 ± 0.31	4.30 ± 0.26	4.27 ± 0.24	0.828
Hb (g dL^−1^)	5.56 ± 0.77	7.03 ± 0.43	6.00 ± 0.54	6.10 ± 0.53	6.87 ± 0.41	0.423
Hct (%)	16.67 ± 1.35	21.00 ± 0.76	18.00 ± 0.94	19.00 ± 1.09	20.67 ± 0.71	0.505
WBC (×10^4^ cells µL^−1^)	4.63 ± 2.06	2.63 ± 0.90	4.63 ± 1.97	2.70 ± 0.62	2.10 ± 0.68	0.579
BUN (mg dL^−1^)	42.97 ± 1.14	48.50 ± 0.81	55.17 ± 1.36	50.43 ± 1.54	53.67 ± 1.91	0.263
Creatinine (mg dL^−1^)	0.07 ± 0.02	0.06 ± 0.01	0.05 ± 0.01	0.07 ± 0.04	0.06 ± 0.01	0.712
Cholesterol (mg dL^−1^)	32.00 ± 1.23	34.33 ± 1.35	37.00 ± 1.42	34.33 ± 1.25	39.67 ± 1.53	0.549
Triglyceride (mg dL^−1^)	34.33 ± 2.97	30.33 ± 1.50	51.00 ± 2.04	45.00 ± 2.67	41.67 ± 2.97	0.739
HDL-Cholesterol (mg dL^−1^)	12.67 ± 0.71	12.33 ± 0.98	12.00 ± 0.75	11.33 ± 0.63	13.00 ± 0.57	0.814
LDL-Cholesterol (mg dL^−1^)	12.33 ± 1.08	16.00 ± 0.81	15.33 ± 1.00	14.00 ± 0.58	18.33 ± 1.30	0.436
Total protein (g dL^−1^)	1.67 ± 0.28	1.80 ± 0.18	1.87 ± 0.19	1.86 ± 0.22	1.97 ± 0.19	0.245
Blood performance *	54.47 ± 3.22	56.49 ± 7.79	54.74 ± 1.29	54.46 ± 3.77	50.46 ± 7.53	0.961
AST (U L^−1^)	75.33 ± 2.18	97.67 ± 3.31	111.67 ± 2.88	92.00 ± 1.20	131.67 ± 5.27	0.593
ALT (U L^−1^)	3.40 ± 0.59	2.67 ± 0.40	4.00 ± 0.01	3.33 ± 0.43	3.90 ± 0.94	0.751
ALP (U L^−1^)	344.33 ± 7.81	308.00 ± 5.21	345.67 ± 3.96	364.67 ± 6.10	399.00 ± 8.23	0.949

1M12, one meal daily at 12.00 h; 2M8–12, two meals daily at 08.00 and 12.00 h; 2M8–16, two meals daily at 08.00 and 16.00 h; 2M12–16, two meals daily at 12.00 and 16.00 h; 3M8–12–16, three meals daily at 08.00, 12.00 and 16.00 h; RBC, red blood cells; Hb, hemoglobin; Hct, hematocrit; WBC, white blood cells; BUN, blood urea nitrogen; HDL, high-density lipoprotein; LDL, low-density lipoprotein; AST, aspartate aminotransferase; ALT, alanine aminotransferase; ALP, alkaline phosphatase; * Blood performance was calculated from RBC (×10^6^ cells µL^−1^) + Hct (g dL^−1^) + WBC (×10^3^ cells µL^−1^) + total protein (g dL^−1^), as reported by Montazeri Parchikolaei [31]; Data are expressed as tank mean ± SEM; Differences between means were tested with Duncan’s multiple range test; Different superscripts in the same row indicate a significant difference (*p* < 0.05).

## Data Availability

Data available on request from the authors.
